# Incidence and Prognostic Relevance of Cardiopulmonary Failure in Takotsubo Cardiomyopathy

**DOI:** 10.1038/s41598-017-15327-3

**Published:** 2017-11-07

**Authors:** Ibrahim El-Battrawy, Siegfried Lang, Uzair Ansari, Katherine Sattler, Michael Behnes, Katja Schramm, Christian Fastner, Erol Tülümen, Xiaobo Zhou, Ursula Hoffmann, Martin Borggrefe, Ibrahim Akin

**Affiliations:** 1 First Department of Medicine, Faculty of Medicine, University Medical Centre Mannheim (UMM), University of Heidelberg, Mannheim, Germany; 2 DZHK (GermanCenter for Cardiovascular Research), Partner Site, Heidelberg-Mannheim, Mannheim, Germany; 3 Key Laboratory of Medical Electrophysiology of Ministry of Education, Institute of Cardiovascular Research, Southwest Medical University, Luzhou, Sichuan China

## Abstract

Recent studies have indicated that patients with takotsubo cardiomyopathy (TTC) have a higher mortality rate than the general population. There is a distinct possibility that TTC could be associated with adverse life-threatening complications like cardiopulmonary failure. Our institutional database constituted a collective of 114 patients diagnosed with TTC. The frequency, determinants and predictors of cardiopulmonary failure were assessed. The patients were subsequently classified into two groups based on the presence (n = 44, 38.6%) or absence (n = 70, 61.4%) of cardiopulmonary failure. Multivariable logistic-regression analysis identified impaired left ventricular function defined as ≤35% at presentation and life-threatening arrhythmia as a positive significant independent predictor of cardiopulmonary failure. A majority of the patients with cardiopulmonary failure were treated with either non-invasive or invasive ventilator support (88%), while 48% of the patients required treatment with catecholamine. The in-hospital mortality rate was greater in the cardiopulmonary failure group. Cardiopulmonary failure patients were at ongoing increased risk of death with a higher mortality at 30-day, 1-year and at 5 years of follow-up. Cardiopulmonary failure is a frequent complication in TTC with an increased short- and long-term mortality. Patient susceptible to this condition could be identified by a reduced ejection fraction and life-threatening arrhythmia.

## Introduction

Takotsubo cardiomyopathy (TTC), first described 1990, is a transient disorder of ventricular wall dysfunction characterized by a range of wall motion abnormalities and clinically representative of an acute heart failure syndrome with substantial risk for adverse events^1–3^. Recent data has attempted to classify TTC into four different forms based on the region of manifestation^4^. The apical form is the most common (81.7%) followed by the mid-ventricular (14.6%), basal (2.2%) and the focal form (1.5%). The exact pathophysiological mechanism for selective wall motion abnormality in the absence of significant coronary artery stenosis remains unknown. Patients present with symptoms such as angina pectoris, which may mimic an acute coronary syndrome (ACS). TTC may also be associated with some critical complications such as cardiopulmonary failure, life-threatening arrhythmias, atrial fibrillation, acquired long QTs, thromboembolic events and cardiac rupture^2–6^. There is lack of data describing the incidence and clinical impact of cardiopulmonary failure in TTC patients. The present study was conducted to determine the short-term and long-term prognostic impact of cardiopulmonary failure diagnosed in patients suffering from TTC.

## Methods

We retrospectively studied a collective of 114 consecutive patients diagnosed with TTC between January 2003 and September 2015 at our institution. Patients were diagnosed according to the Mayo Clinic Criteria^7^, which outlines the clinical features associated with TTC.

These criteria essentially highlight the transient wall motion abnormality in the left ventricular associated with or without an apical involvement; mention regional wall motion abnormalities that extend beyond a single epicardial vascular distribution; and also describe an event that occurs frequently, but not always in the wake of a stressful trigger. Other salient points mandate the absence of obstructive coronary disease; focus on the appearance of new ECG pathologies, which mimic ACS or modest elevations in cardiac troponin levels; and also underline the absence of pheochromocytoma or myocarditis in the patient. Ballooning pattern was defined according to defined criteria being a transient systolic dysfunction with marked LV contraction abnormality due to akinesia or dyskinesia of the LV apical and/or midventricular or basal segments extending beyond a single coronary perfusion bed^8,9^. Ballooning pattern was defined using LV-angiography and echocardiography and in uncertain cases cardiac MRI was done.

The angiograms, echocardiograms and ECGs were reviewed by two experienced independent cardiologists to evaluate the diagnosis of TTC. The study protocol was approved by the Ethics Committee of University Medical Centre Mannheim. The need for informed consent was waived by the ethics committee. All methods were performed in accordance with the relevant guidelines and regulations.

In-hospital events, arrhythmias, cardiac rupture, thromboembolic events, pulmonary congestion with the use of non-invasive positive-pressure ventilation, endotracheal intubation, use of a temporary pacemaker, use of catecholamines, and in-hospital death were assessed based on chart review. The primary end point of our study was the all-cause mortality of TTC as assessed by chart review and/or telephonic interview. If medical records, treating physicians or relatives were unable to substantiate information identifying the cause of death, it was noted as death due to an unknown cause.

All patients presenting with acute heart failure and/or cardiogenic shock were grouped together as suffering from some form of cardiopulmonary failure. An acute heart failure was defined by the presence of pulmonary edema in a patient requiring non-invasive and/or invasive mechanical support while cardiogenic shock was diagnosed in a patient with a sustained systolic blood pressure <90 mmHg and presenting with signs of tissue hypoperfusion, essentially requiring treatment with catecholamines.

### Statistics

Data are presented as means ± SD for continuous variables with a normal distribution, median (interquartile range) for continuous variables with a non-normal distribution, and as frequency (%) for categorical variables. The Kolmogorov–Smirnov test was used to assess normal distribution. Student’s t-test and the Mann–Whitney U-test were used to compare continuous variables with normal and non-normal distributions, respectively. The Chi-squared-test or Fisher’s exact test was used to compare categorical variables. The log-rank test was used to compare the survival curves between the cardiopulmonary failure group and the non-cardiopulmonary failure group. Factors with p < 0.10 on univariate analysis were entered into the Cox multivariate regression to define independent risk factors for the end-point. Statistical analysis was performed with SPSS 23.0 in all analyses, p ≤ 0.05 (two-tailed) was taken to indicate statistical significance.

## Results

### Baseline demographics

We studied clinical and echocardiographic characteristics in 114 TTC patients with a mean follow-up of 1529 ± 1121 days. Table 1 summarizes this data with a predominance of postmenopausal females in both groups. Patients with cardiopulmonary failure were younger, and required longer duration of care in the intensive unit as compared to TTC patients without cardiopulmonary failure. Although lower ejection fraction (EF) values, with Simpson’s method, were recorded in the cardiopulmonary failure group, the subsequent recovery of EF to normal range was observed in both groups. The ballooning pattern at presentation on echocardiography was also similar in both groups.

**Table 1:** Baseline characteristics of 114 patients initially presenting with TTC.

Variables	No-cardiopulmonary failure (n = 70)	Cardiopulmonary failure (n = 44)	p value*
**Demographics**			
Age, mean ± SD	69 ± 10.5	64 ± 11.8	**0**.**02**
Female, n (%)	61 (87.1)	34 (77.3)	0.20
**Symptoms**, **n** (**%**)			
Dyspnoe	22 (31.4)	21 (47.7)	**0**.**08**
Chest pain	39 (55.7)	19 (43.1)	0.14
**Clinic parameter**			
Systolic BP, mmHg	140 ± 26	119 ± 36	**<0**.**01**
Diastolic BP, mmHg	80 ± 13	69 ± 22	**<0**.**01**
Heart rate, bpm	96 ± 28	107 ± 27	**0**.**04**
**ECG Data**, **n (%)**			
ST-segment elevation	19 (27.1)	15 (34)	0.43
Inversed T-Waves	62 (88.5)	40 (91)	0.66
QTc (ms), mean ± SD	486 ± 55.5	468 ± 45	**0**.**07**
QRS (ms), mean ± SD	84.3 ± 13.6	88.3 ± 16.3	0.20
**Stress factor**, **n (%)**			
Emotional sress	26 (37.1)	4 (9)	**<0**.**01**
Physical stress	35 (50)	29 (66)	**0**.**09**
None	14 (20)	11 (25)	0.53
**Laboratory values**, **mean ± SD**			
Troponin I (U/L)	3.3 ± 4.5	5.4 ± 7.1	**0**.**08**
Creatine phosphatkinase (U/L)	401 ± 923	1051 ± 4093	0.21
C-Reactive protein (mg/l)	39 ± 66	64 ± 95	0.12
Creatinine (mg/dl)	1.1 ± 0.81	1.1 ± 0.54	0.97
**Echocardiography data, n (%)**			
LV EF %	40 ± 9	35 ± 9	**<0**.**01**
Right ventricular involvement	14 (20)	12 (27.2)	0.36
Apical type	49 (70)	33 (75)	0.50
Tricuspid regurgation	32 (45.7)	17 (38.6)	0.45
Mitral regurgation	43 (61.4)	17 (38.6)	**0**.**01**
**Medical history**, **n (%)**			
Smoking	21 (30)	15 (34)	0.64
Diabetes mellitus	26 (22.8)	5 (44)	0.98
BMI > 25 kg/m^2^	24 (47.3)	7 (18)	**<0**.**01**
Hypertension	43 (61.4)	23 (52.3)	0.33
COPD	14 (20)	8 (18)	0.81
History of malignancy	9 (13)	7 (16)	0.64
**Drugs on admission**, **n (%)**			
Beta-blocker	25 (35.7)	10 (22.7)	0.13
ACE inhibitor	22 (31.4)	13 (29.5)	0.85
ARB	9 (13)	2 (4.5)	0.19
Statin	10 (14.3)	9 (20.4)	0.37
Aldosterone antagonist	1 (1.4)	0 (0)	1.00

*p values for the comparison between cardiopulmonary failure and no cardiopulmonary failure; SD, Standard deviation; ECG, Electrocardiogram; EF, Ejection fraction; BMI, body-mass-index, COPD, Chronic obstructive pulmonary disease; ACE, Angiotensin-convetring-enzyme; ARB, Angiotensin-receptor blocker.

### Incidence of cardiopulmonary failure

Cardiopulmonary failure was diagnosed in 44 patients (38.6%). Univariable cox-regression analysis identified age, atrial fibrillation, life-threatening arrhythmia, QTc and EF ≤ 35% as predictors for the development of cardiopulmonary failure (Table 2). Multivariable logistic-regression analysis identified impaired left ventricular function defined as ≤35% at presentation (OR 3.9, 95% CI 1.5–10.1; p < 0.01) and life-threatening arrhythmia (OR 7.3, 95%CI 1.2–43.7; p = 0.03) as a positive significant independent predictor of cardiopulmonary failure.

**Table 2:** Predictors of cardiopulmonary failure.

	Univariate analysis			Multivariate analysis		
	OR	95%CI	P-value	OR	95%CI	P-value
Male	1.9	0.7–5.3	0.17			
Age	0.9	0.9–1.0	**0**.**02**	0.9	0.9–1.0	**0**.**01**
Apical ballooning	1.3	0.5–3.2	0.50			
Atrial fibrillation	2.5	0.9–6.6	**0**.**05**	2.2	0.6–8.6	0.22
Life-threatening arrhythmia	11.3	2.3–54.1	**<0**.**01**	7.3	1.2–43.7	**0**.**03**
EF ≤ 35%	3.8	1.7–8.4	**<0**.**01**	3.9	1.5–10.1	**<0**.**01**
DM Typ II	0.9	0.4–2.4	0.98			
GFR < 60 ml/min	1.0	0.4–2.4	0.91			
History of cancer	1.3	0.4–3.7	0.64			
QTc	1.0	0.9–1.0	**0**.**08**	0.9	0.9–1.0	0.31

HR, hazard ratio; EF, ejection fraction; CRP, c-reactive protein; GFR, glomerular filtration rate.

### Clinical course and treatment strategy

Cardiopulmonary resuscitation was necessary in 9 of the patients (7.9%) from the general TTC population. Life-threatening arrhythmias were more observed patients (n = 11; 25%) with cardiopulmonary failure compared with patients (n = 2; 2.8%) without cardiopulmonary failure; p < 0.01.

The cardiopulmonary resuscitation was performed either out-of-hospital and before admission (n = 2) or during the hospital stay (n = 7). The reasons for cardiopulmonary resuscitation were: asystole (n = 4), ventricular tachycardia as well as torsade de pointes (n = 6), ventricular fibrillation (n = 4) and complete AV-block (n = 1). Recurrent life-threatening arrhythmias during in-hospital stay were documented in only 2 patients.

A single patient required treatment with a pacemaker, while two other patients received an implantable defibrillator and they belonged to the cardiopulmonary failure group. Mechanical circulatory support systems such as the intraaortic ballon pump (IABP, n = 1) or extracorporal membrane oxygenation (ECMO; n = 2) were used in 8.8% of TTC patients with cardiopulmonary failure. (Table 3) 48% of patients were treated with catecholamines.

**Table 3:** Clinical course and treatment strategy.

Variables	Non-cardiopulmonary failure (n = 70)	Cardiopulmonary failure (n = 44)	p value*
Life-threatening arrhythmia, n (%)	2 (2.8)	11 (25)	**<0**.**01**
Cardiopulmonary resuscitation, n (%)	1 (1.4)	8 (18)	**<0**.**01**
IABP, n (%)	0 (0)	1 (1)	1.000
Veno-arterial-ECMO, n (%)	0 (0)	2 (4.6)	0.146
Admission to ICU, length of stay	2.4 ± 1.6	7.5 ± 9	**<0**.**01**
In-hospital death, n (%)	1 (1.4)	8 (18)	**<0**.**01**
Thromboembolic events, n (%)	8 (11.4)	6 (13.6)	0.72
Acquired Long QTs, n (%)	44 (63)	29 (66)	0.93

*p values for the comparison between classical group and adverse events group; ECMO, Extracorporal membrane oxygenation; IABP, Intraaortic balloon pump; ICU, Intermediate care unit.

### Short- and long-term outcome

**Figure 1:**
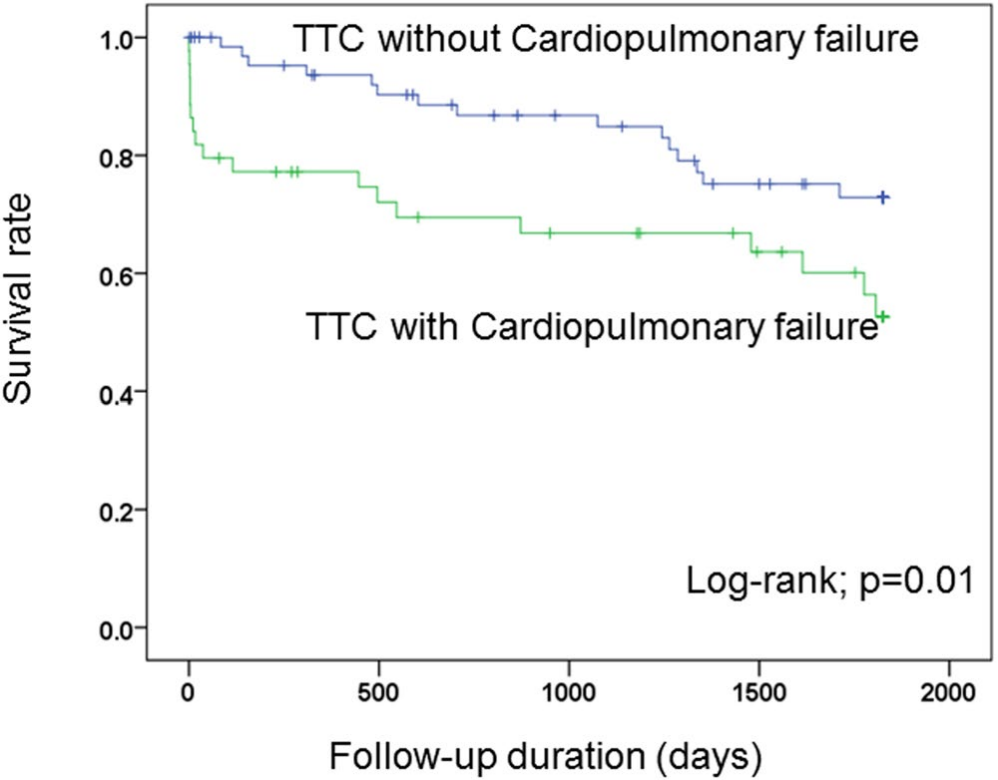
Kaplan-Meier curve shows lower short- and long-term mortality rate in absence of cardiopulmonary failure over 5 years follow-up.

In TTC patients suffering from cardiopulmonary failure, the 30-day mortality was significantly higher as compared to patients without cardiopulmonary failure (19% versus 10%, p < 0.05). Additionally, patients with cardiopulmonary failure showed an ongoing increased risk of death over a 30-day (18% versus 1.4%; p < 0.01), 1-year (22.7% versus 5.7%; p < 0.05) and 5-year (41.0% versus 21.4%; p < 0.05); Table 4, Fig. 1. Patients, who died at this period required a longer duration of care in the intensive unit (6.7 ± 9.6 days versus 3.3 ± 3.4 days; p = 0.09) and demonstrated a significantly lower EF as compared to patients without cardiopulmonary failure (34 ± 8.5% versus 40.3 ± 9.3%; p < 0.01).

**Table 4:** Outcome in TTC with and without cardiopulmonary failure.

Variables	No-cardiopulmonary failure (n = 70)	Cardiopulmonary failure (n = 44)	Relative risk (95%CI)	p value*
In-hospital mortality, n (%)	1 (1.4)	8 (18)	5.9 (0.92 37.47)	**<0**.**01**
30-Day mortality, n (%)	1 (1.4)	8 (18)	5.9 (0.92–37.47)	**<0**.**01**
1-Year mortality, n (%)	4 (5.7)	10 (22.7)	2.3 (0.99–5.35)	**<0**.**05**
5-Year mortality, n (%)	15 (21.4)	18 (41)	1.5 (0.99–2.23)	**<0**.**05**
Cardiovascular mortality, n (%)	2 (2.8)	9 (25)	3.6 (1.03–12.82)	**<0**.**01**
Non-cardiovascular mortality, n (%)	13 (18.5)	9 (25)	1.0 (0.71–1.3)	0.80

*p values for the comparison between cardiopulmonary failure and non-cardiopulmonary failure group; Date are presented as number (%), CI: confidence interval.

A cardiovascular cause for death was more pronounced in patients with cardiopulmonary failure as compared to the other patient group. (25.0% versus 2.8%; p < 0.01). In Cox univariate analysis male gender (p = 0.01), CRP (p < 0.01), glomerular filtration rate (GFR) < 60 ml/min (p = 0.01), Troponin I (p = 0.04), EF ≤ 35% (p < 0.01), shock (p < 0.01) and the use if catecholamines (p < 0.01) were associated with the primary end point. In multivariate Cox regression analysis the EF ≤ 35% (HR 7.2; 95%CI 1.4–36.0; p = 0.01) and GFR < 60 ml/min. (HR 2.5; 95%CI 1.0–11.0; p = 0.03) figured out as an independent predictor of the primary endpoint, Table 5.

**Table 5:** Multivariate analysis for the end point.

	Univariate analysis			Multivariate analysis		
	HR	95%CI	P-value	HR	95%CI	P-value
Male	2.6	1.2–5.7	**0**.**01**	2.0	0.3–13.0	0.43
CRP	1.0	1.0–1.0	<**0**.**01**	1.0	0.9–1.0	0.13
GFR < 60 ml/min	2.5	1.2–5.1	**0**.**01**	3.5	1.0–11.0	**0**.**03**
Troponin I	1.1	1.0–1.2	**0**.**04**	1.0	0.9–1.2	0.26
Shock	4.1	2.0–8.4	<**0**.**01**	11.3	0.5–24.2	0.12
EF ≤ 35%	4.8	2.2–104	<**0**.**01**	7.2	1.4–36.4	**0**.**01**
QRS duration (ms)	1.0	0.9–1.0	0.40			
Emotionalerstress	0.4	0.1–1.1	0.10			
Catecholamines	3.9	1.9–7.9	<**0**.**01**	0.06	0.0–1.0	0.06
DM Typ II	1.0	0.7–1.4	0.81			
Hypertension	0.9	0.7–1.2	0.64			
Apical ballooning	1.1	0.8–1.4	0.39			
History of cancer	1.7	0.7–4.2	0.21			
Smoking	0.7	0.3–1.6	0.49			

HR, hazard ratio; EF, ejection fraction, CRP, C-reactive protein; GFR, glomerular filtration rate.

## Discussion

We conducted a retrospective clinical investigation in 114 TTC patients, and could postulate that (i) the incidence of cardiopulmonary failure in TTC is higher than expected; (ii) the in-hospital morbidity and mortality rates were significantly higher in the cardiopulmonary failure TTC subgroup; (iii) the short-term and long-term prognosis was poorer in TTC patients presenting with cardiopulmonary failure at index-event; (iiii) EF ≤ 35% at admission and life-threatening arrhythmia might be independent predictors for this complication.

### Frequency and predictors of cardiopulmonary failure

TTC was initially thought to be a transient disorder of the heart with a good prognosis. New studies confirm that the mortality rate among TTC patients ranges between 1% and 8%, and comparable to patients suffering from an acute coronary syndrome^10–15^.

Our study, perhaps the first in published medical literature, documents and compares in-hospital complications as well as elucidates the short and long-term prognosis of cardiopulmonary failure among TTC patients. This inherently includes the spectrum of patients further sub-classified as presenting with cardiogenic shock and/or acute heart failure with need of mechanical ventilator support and/or catecholamine support.

The incidence of cardiogenic shock in TTC populations is numbered to be between 2.8% and 12.4%^3,5,16–19^. In our study, the incidence of cardiogenic shock and acute heart failure with need of mechanical respiratory support in TTC patients was between 18.4% and 35%, which is significantly higher than that reported in available literature^3,5,7,16–22^. A lower left ventricular EF has been identified as a significant determinant of cardiopulmonary failure in TTC patients. These findings concur with other studies, which demonstrate that a highly reduced left ventricular EF is an independent predictor of cardiogenic shock with higher mortality rates among TTC patients^18^.

### Treatment strategies in TTC complicated by cardiopulmonary failure

Lack of data pertaining to management strategies for TTC patients in the setting of acute heart failure, has laid foundation to an individualized regimen of drug and respiratory support. Although the basic tenets of management are similar to the therapy of an uncomplicated acute heart failure, the treatment of cardiopulmonary failure in the setting of TTC can be particularly challenging because catecholamine use, predicated in cardiogenic shock, may worsen the clinical course given the presumed causal association between increased catecholamine levels and the occurrence of TTC^23–25^. Additionally, patients with acute respiratory failure requiring mechanical ventilation also have an inherent risk to develop TTC^26^. This is explained by the observation that patients requiring mechanical ventilation have higher levels of catecholamines, which has been implicated in the pathophysiology of TTC. It is believed that these catecholamines may play a role in myocardial stunning, epicardial coronary arterial spasm, microvascular dysfunction and direct myocyte injury^22,24^.

Therefore, the need for alternative circulatory support in cardiogenic shock among TTC patients has been evaluated. A small study investigated the calcium sensitizer levosimendan in a series of 13 TTC patients. A few case reports have also demonstrated the successful use of levosimendan and the phosphodiesterase-3-inhibitor milirinone in TTC cases complicated with cardiogenic shock^16,20,27,28^. In addition, the use of active extracorporeal membrane oxygenator (ECMO) or passive IABP has been reported in several study populations and single case reports. This might reduce the dependence on catecholamines, thus improving the clinical course of cardiogenic shock in TTC. In our study, most patients were treated with catecholamines while Veno-arterial-ECMO and IABP was used in three patients. Respiratory support was provided with non-invasive positive pressure support and/or invasive positive pressure. The influence of alternative respiratory support such as high-flow nasal cannula oxygen therapy could not be established in this study and is an area requiring further evaluation. Recent studies have shown that high-flow therapy via nasal cannula has a positive outcome in acute heart failure^29,30^.

### Outcome in patients with cardiopulmonary failure in TTC

Our initial research indicated that there is lack of data describing the long-term outcome of patients with cardiopulmonary failure complicating TTC. Nevertheless, current literature provides evidence underlining a high short- and long-term mortality (66.7%) over a maximal follow-up of 3.6 years in TTC complicated with cardiogenic shock^18^. Stiermaier *et al*. reported that the mortality rate increased from 61%, 1-year after the index event to 66% after 3.6 years of the index event. Our study is the first of its kind, in evaluating the short- and long-term prognosis of cardiopulmonary failure in TTC over a mean follow-up period of five years. In our analysis, the one-year mortality was about 22.7%. This increased to 41% over a mean follow-up of five years and a cardiovascular cause of death was predominant among these patients. Interesting to note also was the fact that adverse events such as cardiogenic shock and/or respiratory failure have a greater than expected impact on the long term-prognosis in TTC patients. Our study provides new insights into the prognosis of patients with adverse events in TTC and recommend that the patient group complicated with cardiopulmonary failure need a closer follow-up after discharge.

### Study limitations

Our study had some limitations; firstly, this was a single-centre retrospective observational study admitting patients diagnosed over a period of 13 years. Secondly, the use of catecholamines and mechanical support in majority of the patients did not allow the assessment of the prognostic impact of different treatment strategies such as VA-ECMO, IABP and high-flow therapy via nasal cannula. Furthermore, there was no standardized treatment strategy of patients suffering from cardiopulmonary failure.

## Conclusions

The incidence of cardiopulmonary failure in patients with TTC is surprisingly high. Decreased EF and life-threatening arrhythmia might be predictors for cardiopulmonary failure. The rate of in-hospital mortality and long-term mortality were higher in this patient population as compared to those not suffering from cardiopulmonary failure. These results provide new insights to the prognosis of patients diagnosed with TTC.
